# Diet-induced obesity alleviates epithelial damage in hyperoxic acute lung injury (HALI) in mice

**DOI:** 10.1186/s12931-026-03663-w

**Published:** 2026-04-09

**Authors:** Morten Kampelmann, Andreas Pich, Christian Mühlfeld, Julia Schipke

**Affiliations:** 1 Institute of Functional and Applied Anatomy, Hannover Medical School, Carl-Neuberg-Str. 1, Hannover, 30625 Germany; 2 German Center for Lung Research (DZL), Biomedical Research in Endstage and Obstructive Lung Disease Hannover (BREATH), Hannover, Germany; 3 Institute of Toxicology, Hannover Medical School, Hannover, Germany; 4 Core Facility Proteomics, Hannover Medical School, Hannover, Germany

**Keywords:** Hyperoxic acute lung injury, Diet-induced obesity, Obesity paradox

## Abstract

**Background:**

Oxygen therapy is often lifesaving for critically ill patients with acute respiratory distress syndrome (ARDS). However, high oxygen doses may cause hyperoxic acute lung injury (HALI). In line with the obesity pandemic, numbers of ARDS patients with obesity are rising. Epidemiological data suggest higher morbidity but lower mortality in obese ARDS patients. However, it is currently unclear whether there is a biological basis for this “obesity paradox”. This study used a controlled animal model to investigate influences of diet-induced obesity on HALI-associated structural, molecular and functional changes of the lung.

**Methods:**

Male C57BL/6N mice were fed either control diet (CD) or high fat diet (HFD) for 30 weeks. A subset of the animals was additionally exposed to normobaric hyperoxia (FiO_2_: 90%; Hyper) for 72 h.

**Results:**

Hyperoxia was associated with reduced blood oxygenation and mechanical alterations indicative of pulmonary stiffening. Body fat depots were depleted in CD-Hyper, but not in HFD-Hyper groups. Morphological hallmarks of HALI including fragmentation and loss of epithelial cells as well as septal edema were significantly alleviated in hyperoxic obese mice. Diet-group specific changes in protein abundances suggested regulation of cellular stress response in CD-Hyper, whereas in HFD-Hyper predominantly metabolic and cell adaptive processes were altered.

**Conclusions:**

Diet-induced obesity did not influence functional measures in the acute phase of hyperoxia but prevented depletion of body fat reserves and mitigated structural lung damage indicating a beneficial impact on regeneration. This supports a biological basis for an obesity paradox in ARDS, and should be taken into account for future individualized prevention and therapy in obese patients.

**Supplementary Information:**

The online version contains supplementary material available at 10.1186/s12931-026-03663-w.

## Background

Oxygen therapy is a frequently applied and often lifesaving treatment for critically ill patients. In addition to lung pathologies, a variety of extrapulmonary etiologies can lead to an acute respiratory distress syndrome (ARDS) with increased oxygen demand [1]. However, depending on the inspirated oxygen fractions (FiO_2_) and duration of therapy, the therapeutically administered oxygen can also cause adverse effects, most notably a hyperoxic acute lung injury (HALI) and exacerbation of a preexisting lung injury [2, 3]. A meta-analysis of more than 16.000 critically ill patients showed that higher FiO_2_ levels are associated with increasing mortality without significant clinical benefits [4].

The pathogenesis of HALI is complex and not yet fully understood. A major factor is generation of supraphysiological amounts of reactive oxygen species (ROS), which not only cause direct cytotoxic effects, but also act as secondary messengers amplifying further cell damage [2]. The activation of molecular pathways (e.g. MAP kinase cascades) induces cell apoptosis, while the formation of inflammasome complexes initiates the recruitment of immune cells and a proinflammatory state [5, 6].

Despite these potentially adverse side effects, oxygen is often used uncritically as a medication in healthcare [3], partly because the focus in clinical practice tends to be on avoiding hypoxic episodes rather than on hyperoxic injuries [7]. Besides chronic lung diseases (e.g. COPD, cystic fibrosis) and musculoskeletal disorders (e.g. kyphoscoliosis) severe obesity (BMI > 40 kg/m2) is also a known risk factor for hypercapnia [7].

Obesity is a global health challenge with an increasing prevalence over the last decades. In 2022, approximately 2.5 billion adults worldwide were overweight (BMI > 25 kg/m2), while 890 million of these were classified as obese (BMI > 30 kg/m2) [8]. The number of patients with overweight and obesity in intensive care units (ICUs) is rising accordingly and accounted for around one-third of all ICU patients already several years ago [9, 10].

Overweight and obesity are correlated with increased total mortality rates and chronic low-grade inflammation [11]. Excessive body weight impacts lung mechanics, resulting in reduced lung volumes and compliance [12]. Moreover, obesity induces pulmonary alterations at a cellular level. Previous studies in rodent models showed an obesity-related thickening of the septal capillary walls and the air-blood barrier (ABB), as well as alterations in surfactant lipid composition and function [13, 14]. Moreover, volumes of interstitial cells, extracellular matrix and lipid droplets are increased in alveolar septa compared with controls [15].

The role of obesity in the development and progression of ARDS remains unclear. Obesity is associated with higher morbidity, but reduced mortality rates for ARDS, a phenomenon often referred to as “obesity paradox” [16–18]. There is an ongoing debate as to whether this is solely attributable to confounding factors such as collider stratification bias or closer monitoring of overweight patients, or whether there is a biological basis for it [19, 20].

Given the pandemic rise of obesity in patients receiving oxygen therapy and with ARDS, there is an urgent need to better understand the cellular and molecular relationship between BMI, pulmonary alterations and patient outcome. High oxygen concentrations cause lung toxicity in various animal species, comparable to that seen in ARDS patients, highlighting the use of controlled animal models in ARDS research [2, 21]. However, existing literature on obesity and oxygen exposure is scarce, results are conflicting and often focus on qualitative structural assessment [22, 23]. This study used a controlled animal model combining long-term high-fat feeding for 30 weeks and a 72 h hyperoxia-exposure. The methodology used included design-based stereology as gold standard for quantitative morphometry [24], lung function assessment and a detailed proteome analysis to investigate how diet-induced obesity influences hyperoxic acute lung injury-associated changes in the pulmonary gas exchange region.

## Methods

### Animal studies

Male C57BL/6 N mice were purchased from Charles River (Sulzfeld, Germany) at an age of 5 weeks. Mice were allocated randomly to receive either control diet (CD, 11 kcal% fat; S3542-E040, ssniff Spezialdiäten, Soest, Germany) or high fat diet (HFD; 60 kcal% fat; S3542-E044, ssniff) after one week of acclimatization. A detailed diet composition was published previously [14]. The animals had ad libitum access to water and food, were housed separately in cages with nesting materials and shelters at controlled temperature conditions (21 ± 2 °C). Body weight and food intake were measured regularly.

**Fig. 1:**
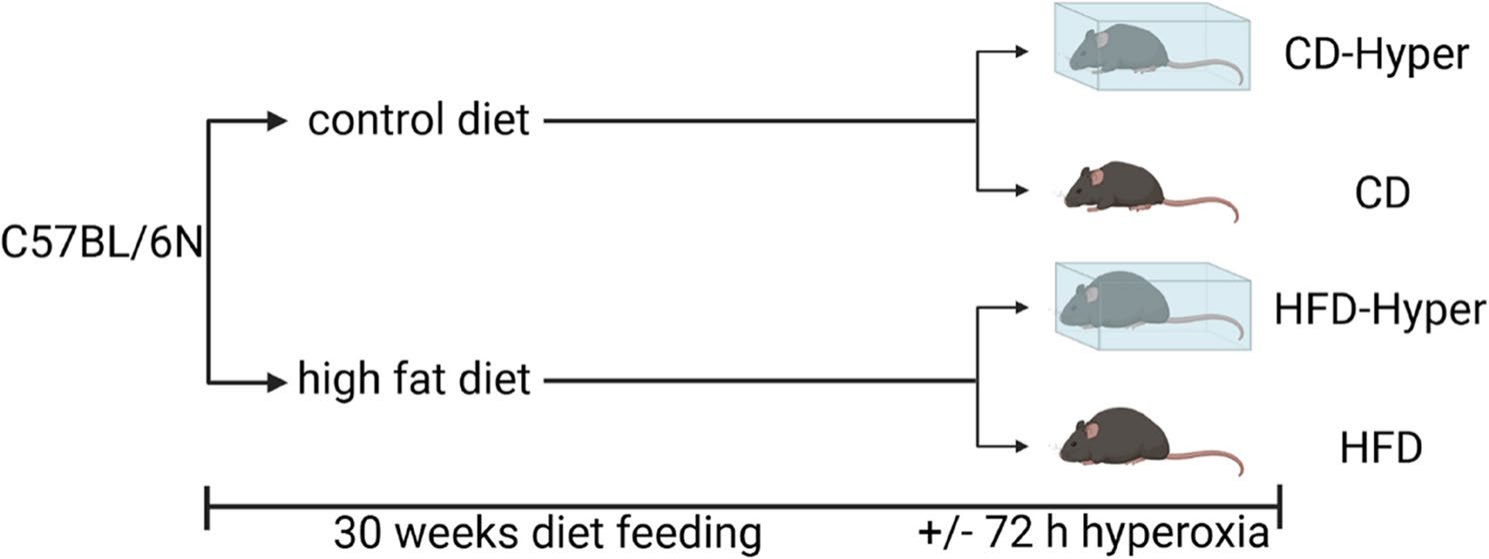
Study design. Control diet, 11 kcal% fat; high fat diet, 60 kcal% fat; hyperoxia, FiO_2_ = 0.9

The total experimental duration was 30 weeks. During the last 7_2_ h, a subset of the animals were exposed to normobaric hyperoxia (Hyper, FiO2 = 0.9) in plexiglas chambers (A-Chamber Animal Cage Enclosure, BioSpherix, Ltd., Parish, NY, USA), resulting in four experimental groups: CD, CD-Hyper, HFD, HFD-Hyper (Fig. 1). The specific n-numbers for the respective experiments are provided in the figure legends. Some animals of CD and HFD groups served as controls in other experimental contexts and were presented before [14, 25, 26].

All animal experiments were permitted and approved by the Lower Saxony State Office for Consumer Protection (LAVES, file number 18/2841) and were conducted in accordance with the European Directive 2010/63/EU.

### Final experiments and organ preparation

After 30 weeks, mice were anesthetized by intraperitoneal injection of ketamine (100 mg/kg body weight; cp. pharma, Burgdorf, Germany) and xylazine (5 mg/kg body weight; Bayer, Leverkusen, Germany). Lung mechanics were assessed with a FlexiVent small animal respirator (SQIREQ, Montreal, QC, Canada) under closed chest conditions and at a positive end expiratory pressure (PEEP) of 3 cmH_2_O. Ambient air was used for ventilation during the measurements. Arterial blood was collected from the tail artery and blood gas analysis was carried out directly afterwards with an iSTAT Alinity device (CG8 + cartridges; Abbott, Wiesbaden, Germany). Finally, the thoracic cavity was opened under sufficient analgesia, right lung lobes were ligated and left lungs were fixed via tracheal instillation with 1.5% paraformaldehyde/1.5% glutaraldehyde in 0.15 M HEPES buffer at a hydrostatic pressure of 20–25 cmH_2_O. Left lungs were incubated in the fixative solution for at least 24 h and further processed for structural analysis as described below. The right lung lobes were snap frozen in liquid nitrogen and stored at -80° C for molecular analyses.

### Structural analyses by design-based stereology

Quantitative structural investigations were performed by design-based stereology in accordance with the recommendation of the American Thoracic Society and European Respiratory Society [24]. The analyst was blinded for the experimental groups during the analysis. Left lung volumes were determined by the water displacement method (Archimedes’ Principle) [27]. Afterwards, systematic uniform random sampling (SURS) of the left lung was performed [28].

For light microscopic (LM) analyses, lung tissue was embedded in glycol methacrylate (Technovit^®^ 7100; Heraeus Kulzer, Wehrheim, Germany) as described previously [29]. Sections with a thickness of 1.5 μm were cut, mounted on glass slides and stained with toluidine blue (Merck, Darmstadt, Germany). Slides were digitized with a slide scanner (AxioScan.Z1, Zeiss, Oberkochen, Germany) and analyzed with the newCast software (Visiopharm, Hørsholm, Denmark). The number of analyzed fields of view was based on the requirement that at least 100 counting events per parameter were assessed. Representative LM images of all experimental groups are shown in the supplement (Fig. S1).

Volumes of parenchyma and non-parenchyma were estimated at a primary magnification of 5x, and a primary magnification of 20x was used for volume estimation of ductal airspaces, alveolar airspaces and alveolar septa, as well as for the septal surface area. The number of AE2 cells was assessed at a primary magnification of 40x with the disector method. For details on probes and calculations please refer to [28, 30].

Samples for transmission electron microscopic (TEM) analyses were subsampled into tissue blocks of ~ 1 mm^3^ size and embedded in epoxy resin (Epon^®^, Serva, Heidelberg, Germany) as described before [29]. 60 nm ultrathin sections were cut, mounted on copper grids and analyzed with a Morgagni 268 microscope (FEI, Eindhoven, Netherlands), equipped with a digital camera (Veleta; Olympus Soft Image Solutions, Münster, Germany). Microscopic images were taken at a primary magnification of 14.000x according to SURS. Representative EM images of all experimental groups are shown in the supplement (Fig. S2). Three blocks per animal and 40 fields of view per block were analyzed using the STEPanizer stereology tool [31]. For details on probes and calculations please refer to [28, 30].

### Sample preparation for MS analysis

The tissue processing for proteome analysis was described in detail previously [14]. In brief, the snap-frozen right lung tissue was homogenized, lysed and centrifuged. The protein content was determined using a Pierce™ BCA kit (Thermo Fischer Scientific, Waltham, MA, USA).

Protein was mixed with Laemmli buffer and incubated for 5 min at 95 °C. Proteins were then alkylated by addition of acrylamide up to a concentration of 2% and incubation at RT for 30 min. SDS PAGE was performed on 12% gels in a mini protean cell (Biorad). After electrophoresis proteins were stained with Coomassie Brilliant Blue (CBB) for 15 min and background staining was reduced with water. Each lane was cut into four pieces which were further minced to 1 mm³ gel pieces. Further sample processing was done as described [32]. Briefly, gel pieces were destained two times with 200 µL 50% ACN, 50 mM ammonium bicarbonate (ABC) at 37 °C for 30 min and then dehydrated with 100% ACN. Solvent was removed and gel pieces were dried in a vacuum centrifuge and 100 µL 10 ng/µL sequencing grade Trypsin (Promega) in 10% ACN, 40 mM ABC were added. Gels were rehydrated in trypsin solution for 1 h on ice and then covered with 10% ACN, 40 mM ABC. Digestion was performed over night at 37 °C and was stopped by adding 100 µL of 50% ACN, 0,1% TFA. After incubation at 37 °C for 1 h the solution was transferred into a fresh sample vial. This step was repeated twice and extracts were combined and dried in a vacuum centrifuge. Dried peptide extracts were redissolved in 30 µL 2% ACN, 0.1% TFA with shaking at 800 rpm for 20 min. After centrifugation at 20,000 x g aliquots of 12.5 µL each were stored at -20 °C.

### LC-MS analysis

Peptide samples were separated with a nano-flow ultra-high pressure liquid chromatography system (RSLC, Thermo Scientific) equipped with a trapping column (3 μm C18 particle, 2 cm length, 75 μm ID, Acclaim PepMap, Thermo Scientific) and a 50 cm long separation column (2 μm C18 particle, 75 μm ID, Acclaim PepMap, Thermo Scientific). Peptide mixtures were injected, enriched and desalted on the trapping column at a flow rate of 6 µL/min with 0.1% TFA for 5 min. The trapping column was switched online with the separating column and peptides were eluted with linear gradient of buffer B (80% ACN, 0.1% formic acid) in buffer A (0.1% formic acid) from 4% to 25% in 30 min, 25% to 50% in 10 min, 50% to 90% in 5 min and 10 min at 90% B. The column was reconditioned to 4% B in 15 min. The Flow rate was 250 nL/min and the column temperature was set to 45 °C. The RSLC system was coupled online via a Nano Spray Flex Ion Soure II (Thermo Scientific) to an LTQ-Orbitrap Velos mass spectrometer. Metal-coated fused-silica emitters (SilicaTip, 10 μm i.d., New Objectives) and a voltage of 1.3 kV were used for the electrospray. Overview scans were acquired at a resolution of 60k in a mass range of m/z 300–1600 in the orbitrap analyzer. The top 10 most intensive ions of charges two or three and a minimum intensity of 2000 counts were selected for CID fragmentation with a normalized collision energy of 38.0, an activation time of 10 ms and an activation Q of 0.250 in the LTQ. Fragment ion mass spectra were recorded in the LTQ at normal scan rate and stored as centroid m/z values. Active exclusion was set for 70 s within a mass window of 10 ppm of the specific m/z value.

Raw MS data were processed using Max Quant software (version 1.5) [33], and a data base containing mouse proteins of uniport (UP000000589) and common contaminants. Proteins were stated identified by a false discovery rate of 0.01 on protein and peptide level.

### Statistics

Statistics of the proteome data was performed with the Perseus software version 1.6.14 [34]. Protein intensities were log_2_-transformed and normalized by median subtraction and missing values were replaced by imputation from normal distribution. Only proteins present in minimum 80% of the animals per cohort were included. Significant different proteins (ANOVA test < 0.05) were used for a principal component analysis (PCA). Two-group comparisons were carried out with a two-sided two-sample student´s t-test without corrections (CD vs. HFD, CD vs. CD-Hyper and CD vs. HFD-Hyper). Proteins with a significant difference (*p* < 0.05) were used for a Venn diagram. Different expression ratios of proteins were visualized by volcano plots. Proteins with -log_10_ *p* values higher than 1.3 and a log_2_-fold change of > 1.00 were indicated in red for upregulated, and proteins with a log_2_-fold change of <-1.00 in blue for downregulated. These up- and downregulated proteins were loaded into the STRING database (version 12.0, https://string-db.org) for enrichment analysis. Detailed results are given in supplemental tables S1-S11.

For all statistical comparisons other than proteome data, Sigma Plot version 13.0 (Systat Software Inc.) was used. *P*-values < 0.05 were considered statistically significant, and *p* values between 0.05 and 0.1 (0.05 < *p* < 0.1) were considered to show a tendency toward significance [35]. Data were tested for normality (Kolmogorov-Smirnov) and equal variance (Brown-Forsythe) and a Two-way ANOVA (2 W-ANOVA) was performed. Exact *p*-values of 2 W-ANOVA analyses are provided in table S12 (Supplemental Material). For interaction effects that were statistically significant or showed a tendency to significance, a post hoc Tukey test was performed afterwards. For correlation analysis, Pearson correlation analysis was performed. Data are shown as means ± standard deviation or as individual values ± standard deviation as indicated in figure legends. Dot Plots were made with GraphPad Prism (version 7), Fig. 1 was created with BioRender.com.

## Results

### Systemic effects of high fat intake and hyperoxia

The high fat diet induced a significant increase in body weight and fat depot weights (Table 1; Fig. S3). Hyperoxia induced a body weight decrease which was more pronounced in CD-Hyper (CD-Hyper vs. CD -6.4%; HFD-Hyper vs. HFD − 3.9%), leading to significantly lower final body weights only in CD-fed hyperoxic animals compared to normoxic controls. In accordance with that, weights of epididymal, retroperitoneal and interscapular fat depots were reduced by half in CD-Hyper compared to CD. This hyperoxia-related reduction in fat reserves was absent in HFD-Hyper animals. The ability to oxygenate the blood, clinically assessed by the Horowitz Index, was significantly diminished in response to hyperoxia, indicating ALI. Here, no diet-dependent differences were observed.

**Table 1:** Systemic effects of diet and hyperoxia

Group	CD	CD-Hyper	HFD	HFD-Hyper	ANOVA results
***n***	7	10	7	10	
Body weight difference [%, W29 vs. finals]	-0.05 ± 3.6	-6.43 ± 3.5	+ 0.07 ± 2.5	-3.90 ± 2.1	Hyperoxia *p* < 0.001
Final body weight [g]	38.1 ± 2.7	31.0 ± 2.8#	50.3 ± 3.5*	48.0 ± 3.2*	Diet *p* < 0.001 Hyperoxia *p* < 0.001 Interaction *p* = 0.034
Fat, epididymal [g]	1.6 ± 0.4	0.8 ± 0.3#	2.4 ± 0.5*	2.1 ± 0.3*	Diet *p* < 0.001 Hyperoxia *p* < 0.001 Interaction *p* = 0.071
Fat, retroperitoneal [g]	0.6 ± 0.2	0.3 ± 0.2#	1.3 ± 0.3*	1.1 ± 0.3*	Diet *p* < 0.001 Hyperoxia *p* = 0.002 Interaction *p* = 0.014
Fat, interscapular [g]	0.4 ± 0.1	0.2 ± 0.1	0.8 ± 0.3	0.8 ± 0.3	Diet *p* < 0.001
Horowitz index [mmHg, pO_2_/FiO_2_]	425.9 ± 97.5	82.7 ± 20.9	468.7 ± 106.9	77.9 ± 18.6	Hyperoxia *p* < 0.001

Shown are group means ± SD. Statistics: 2 W-ANOVA (factors diet and hyperoxia), significant *p*-values or *p*-values showing a tendency to significance are shown on the right. In case of positive interaction effects, pairwise comparisons by post-hoc Tukey test were performed with significant results indicated as: * *p* < 0.05 at the same O_2_ (diet effect); # *p* < 0.05 vs. normoxia in the same diet group (hyperoxia effect) besides the respective values, *W* week

**Fig. 2:**
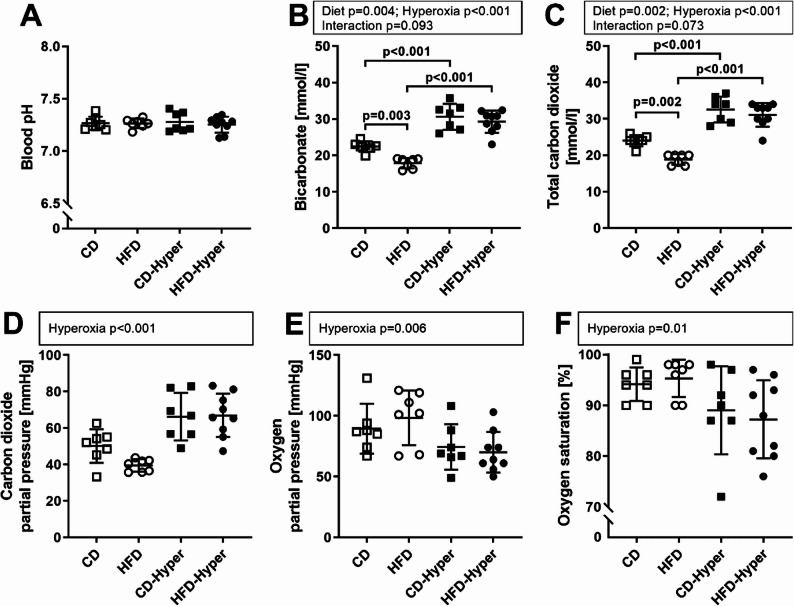
Effects of diet and hyperoxia on arterial blood gases and pH. **A** pH; **B** bicarbonate; **C** total carbon dioxide; **D** carbon dioxide partial pressure; **E** oxygen partial pressure; **F** oxygen saturation. Data are presented as individual values with indicated group means and standard deviations. CD *n* = 7, HFD *n* = 7, CD-Hyper *n* = 7, HFD-Hyper *n* = 9. Statistics: 2 W-ANOVA, significant *p*-values or *p*-values showing a tendency to significance are shown above dot plots. In case of positive interaction effects, pairwise comparisons by post-hoc Tukey Test were performed; *p*-values < 0.05 are indicated

Arterial blood pH values were similar between the groups (Fig. 2A). Bicarbonate and total CO_2_ concentrations were lower in normoxic HFD-fed mice compared to CD-fed animals (Fig. 2B, C). Upon hyperoxia exposure, the partial pressure of carbon dioxide, as well as bicarbonate and total CO_2_ concentrations were elevated, indicating hypoventilation with compensation of a respiratory acidosis (Fig. 2B, C, D). Furthermore, a reduction in partial pressure of oxygen and oxygen saturation in response to hyperoxia was observed (Fig. 2E, F). This was similar in CD-Hyper and HFD-Hyper groups.

### Lung proteome

**Fig. 3:**
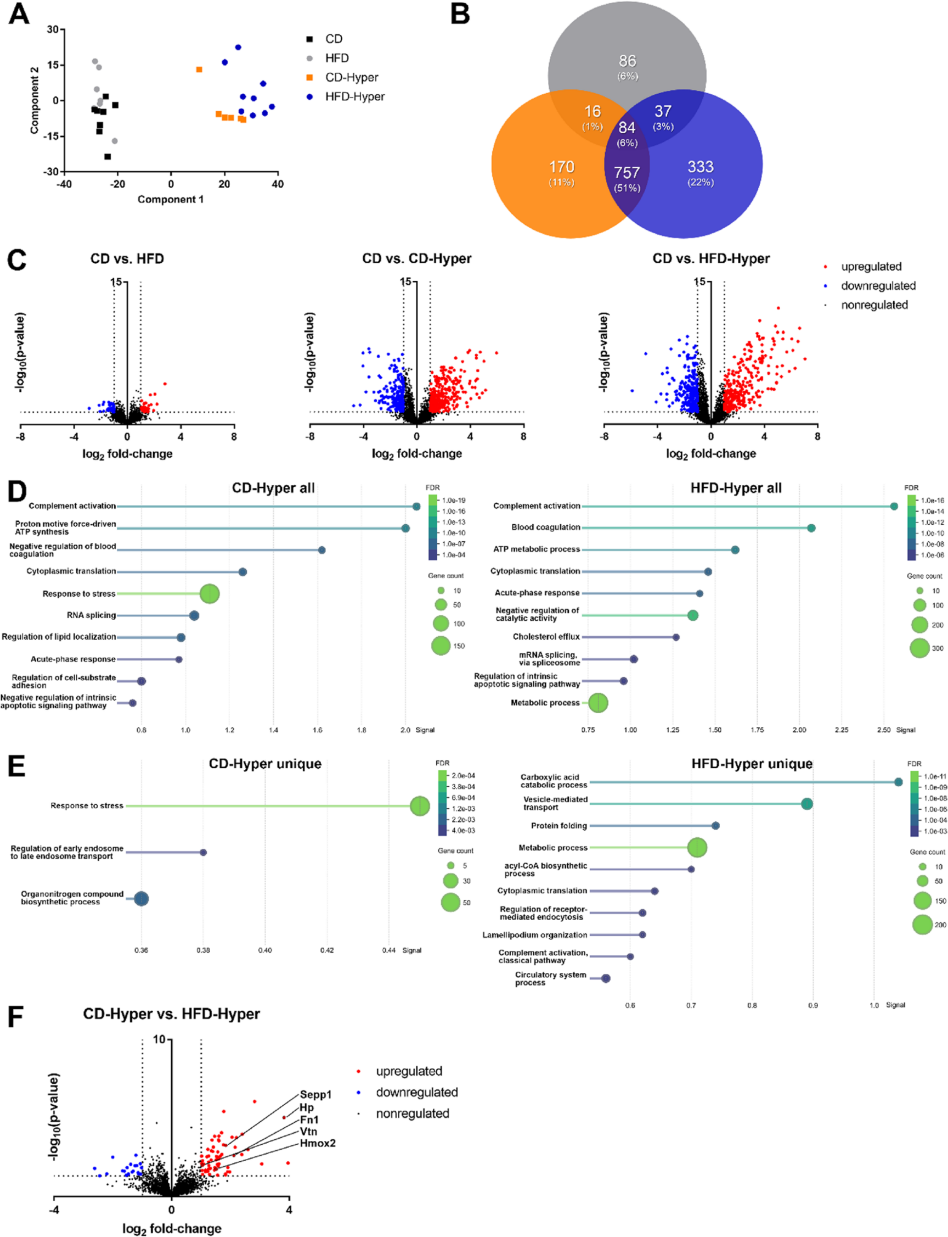
Effects of diet and hyperoxia on the lung proteome. **A** Principal component analysis; **B** Venn diagram of protein changes in comparison to the CD group; **C** volcano plots, dashed lines represent (i) -log10 *p* > 1.301 and (ii) log2-fold change > 1.0 or < 1.0; proteins beyond these thresholds are color-coded in blue for reduced abundance (CD vs. HFD: 36 proteins, CD vs. CD-Hyper: 204 proteins, CD vs. HFD-Hyper: 257 proteins) and in red for increased abundance (CD vs. HFD: 30 proteins, CD vs. CD-Hyper: 287 proteins, CD vs. HFD-Hyper: 295 proteins); **D** pathway analysis by STRING database of differentially expressed proteins in CD-Hyper (left) or HFD-Hyper (right) compared to CD with *p* < 0.05 and log2-fold change > 1.0 or < 1.0, shown are Gene Ontology enrichment biological processes; **E** pathway analysis by STRING database of differentially expressed proteins with *p* < 0.05 that were uniquely altered in CD-Hyper (left) or HFD-Hyper (right) compared to CD, Gene Ontology enrichment biological processes are shown. **F** Volcano Plot for hyperoxic groups, dashed lines represent (i) -log10 *p* > 1.301 and (ii) log2-fold change > 1.0 or < 1.0; proteins beyond these thresholds are color-coded in blue for reduced abundance (22 proteins) and in red for increased abundance (61 proteins). CD *n* = 8, HFD *n* = 7, CD-Hyper *n* = 6, HFD-Hyper *n* = 9

Bulk lung tissue proteome analysis revealed an altered abundance of 223 proteins upon HFD feeding, 1027 proteins upon hyperoxia exposure and 1211 proteins upon the combination treatment (Supplemental Table S1-S3). Principal component analysis indicated that lung proteomes of normoxic CD- and HFD-fed mice had a rather similar composition (Fig. 3A). After hyperoxia exposure, lung proteomes shifted notably and CD-Hyper and HFD-Hyper animals clustered separately, suggesting a diet-dependent adaptation to high oxygen exposure. This was supported by the relatively high number of 170 and 333 proteins uniquely regulated in CD-Hyper and HFD-Hyper, respectively (Fig. 3B).

Significantly altered protein abundances (Fig. 3C) were subjected to pathway analysis with the STRING database. The HFD alone revealed no significantly altered pathways. Hyperoxia exposure induced pathways mainly implicated in complement system and acute phase response, blood coagulation as well as respiratory electron transport chain and ATP synthesis (Fig. 3D, Table S4-S5).

To learn more about the diet-specific adaptation to hyperoxia, uniquely regulated proteins in CD-Hyper and HFD-Hyper were subjected to pathway analysis (Fig. 3E, Table S6-S7). In lungs of the CD-Hyper group, mainly cellular stress response pathways were induced, while in HFD-Hyper metabolic (catabolism of carboxylic acid, acyl-CoA biosynthesis) processes and pathways pointing to cellular adaptation (protein folding, cytoplasmic translation) were predominantly altered. Moreover, the direct comparison of hyperoxic groups revealed a higher abundance of glycoproteins fibronectin 1 and vitronectin, acute phase proteins haptoglobin, as well as the antioxidants selenoprotein P and Heme oxygenase 2 in HFD-Hyper compared to CD-Hyper (Fig. 3F, Table S8). Global comparisons of diet (all CD vs. all HFD) and hyperoxia (all normoxia vs. all hyperoxia) for evaluation of interaction effects are presented in the supplement (Fig. S4, Fig. S5, Table S10, Table S11).

### Lung mechanics and parenchymal composition

**Fig. 4:**
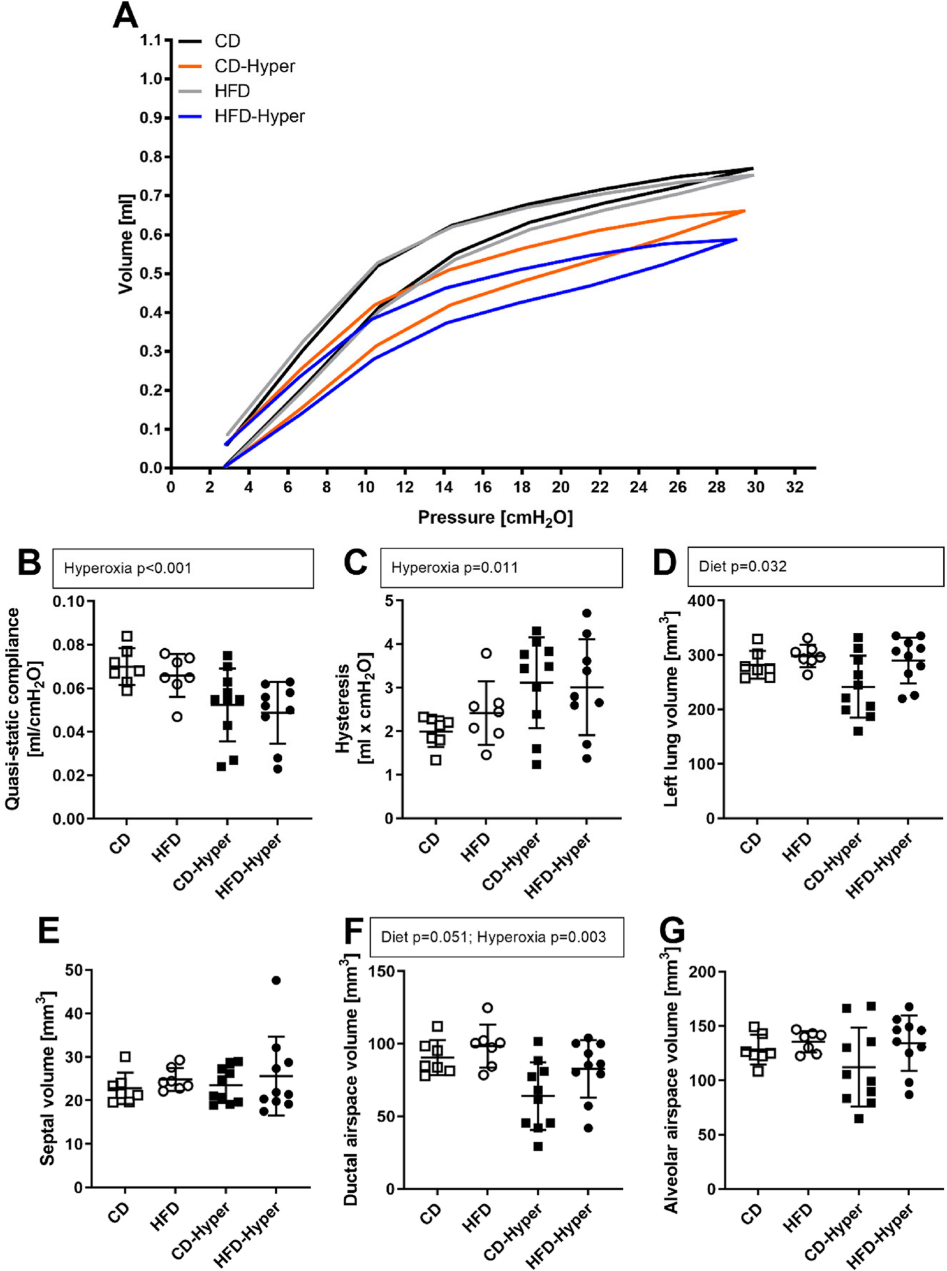
Effects of diet and hyperoxia on lung mechanics and parenchymal composition. **A** Pressure-volume curves; **B** quasi-static lung compliance; **C** hysteresis; **D** left lung volume; **E** septal volume; **F** ductal airspace volume; **G** alveolar airspace volume. Data are presented as means (**A**) or individual values, with indicated group means and standard deviations (**B**-**G**). **A**-**C** CD *n* = 7, HFD *n* = 7, CD-Hyper *n* = 10, HFD-Hyper *n* = 9; **D**-**G** CD *n* = 7, HFD *n* = 7, CD-Hyper *n* = 10, HFD-Hyper *n* = 10. **B**-**G** Statistics: 2 W-ANOVA, significant *p*-values or *p*-values showing a tendency to significance are shown above dot plots. In case of positive interaction effects, pairwise comparisons by post-hoc Tukey Test were performed; *p*-values < 0.05 are indicated

The HFD exerted no obvious effect on lung mechanics but slight increases in left lung and ductal airspace volumes (Fig. 4). Hyperoxia induced a shift of pressure-volume curves and reduced quasi-static lung compliance indicative of a stiffening of the lung (Fig. 4A, B). While septal volumes were not altered by any of the experimental conditions (Fig. 4E), airspace volumes showed a greater variance in hyperoxia-treated animals (Fig. 4F, G). Moreover, ductal airspace volumes were significantly reduced in Hyper groups compared to control conditions (Fig. 4F).

### Structural alterations of the gas exchange region

**Fig. 5:**
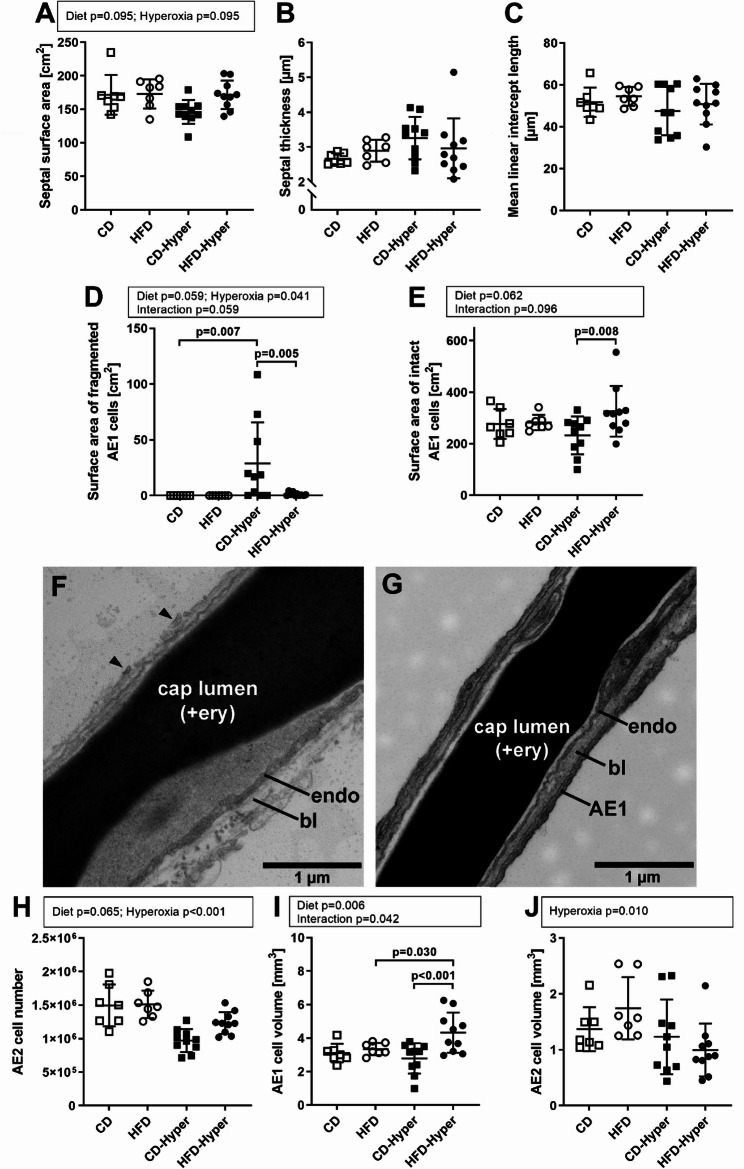
Effects of diet and hyperoxia on the gas exchange region. **A** Septal surface area; **B** septal thickness; **C** mean linear intercept length; **D** surface area of fragmented AE1 cells; **E** surface area of intact AE1 cells. **F** Representative image of septal ultrastructure after hyperoxia exposure, image from CD-Hyper animal; **G** representative image of intact septal ultrastructure, image from CD animal; endo, endothelial cell; bl, basal lamina; AE1, AE1 cell; cap, capillary; ery, erythrocyte; arrowheads in G indicate fragments of epithelial cells. **H** AE2 cell number; **I** AE1 cell volume; **J** AE2 cell volume. Data are presented as individual values, with indicated group means and standard deviations. CD *n* = 7, HFD *n* = 7, CD-Hyper *n* = 10, HFD-Hyper *n* = 10. Statistics: 2 W-ANOVA, significant *p*-values or *p*-values showing a tendency to significance are shown above dot plots. In case of positive interaction effects, pairwise comparisons by post-hoc Tukey Test were performed; *p*-values < 0.05 are indicated

Hyperoxia resulted in several structural alterations within the alveolar region indicative of an acute lung injury, with some distinct diet-dependent differences. The septal surface area was only slightly affected by diet and hyperoxia, the septal thickness was unaltered (Fig. 5A, B). The mean linear intercept length as a surrogate for “wall-to-wall” distances within acinar airspaces was not significantly different, but showed a larger heterogeneity in hyperoxic groups compared to normoxic controls (Fig. 5C). In lean mice, hyperoxia exposure induced a partial fragmentation and detachment of AE1 cells, resulting in a denudation of the basal lamina (Fig. 5D, F; see Fig. 5G for an example of intact septal ultrastructure for comparison). This epithelial damage was much more severe in CD-Hyper compared to HFD-Hyper. Fragmented AE1 cells could be observed on the thin, the thick or on both sides of the air-blood barrier. It should be noted that the sub-individual heterogeneity here was quite high. Consistent with that, the intact AE1 cell surface area decreased in some of the CD-Hyper animals, and increased in some HFD-Hyper mice, respectively (Fig. 5E). Furthermore, hyperoxia induced a reduction in AE2 cell numbers, which was more pronounced in CD-Hyper compared to HFD-Hyper (Fig. 5H; CD: ~1.495 mio AE2 cells ± 0.313 mio; HFD: ~1.511 Mio. cells ± 0.204 mio; CD-Hyper: ~0.969 mio AE2 cells ± 0.171; HFD-Hyper: ~1.234 mio AE2 cells ± 0.163 mio). Hyperoxic HFD-fed mice showed an increase in total AE1 cell volume (Fig. 5I), whereas AE2 cell volumes were decreased in hyperoxic groups (Fig. 5J).

### Lung edema

**Fig. 6:**
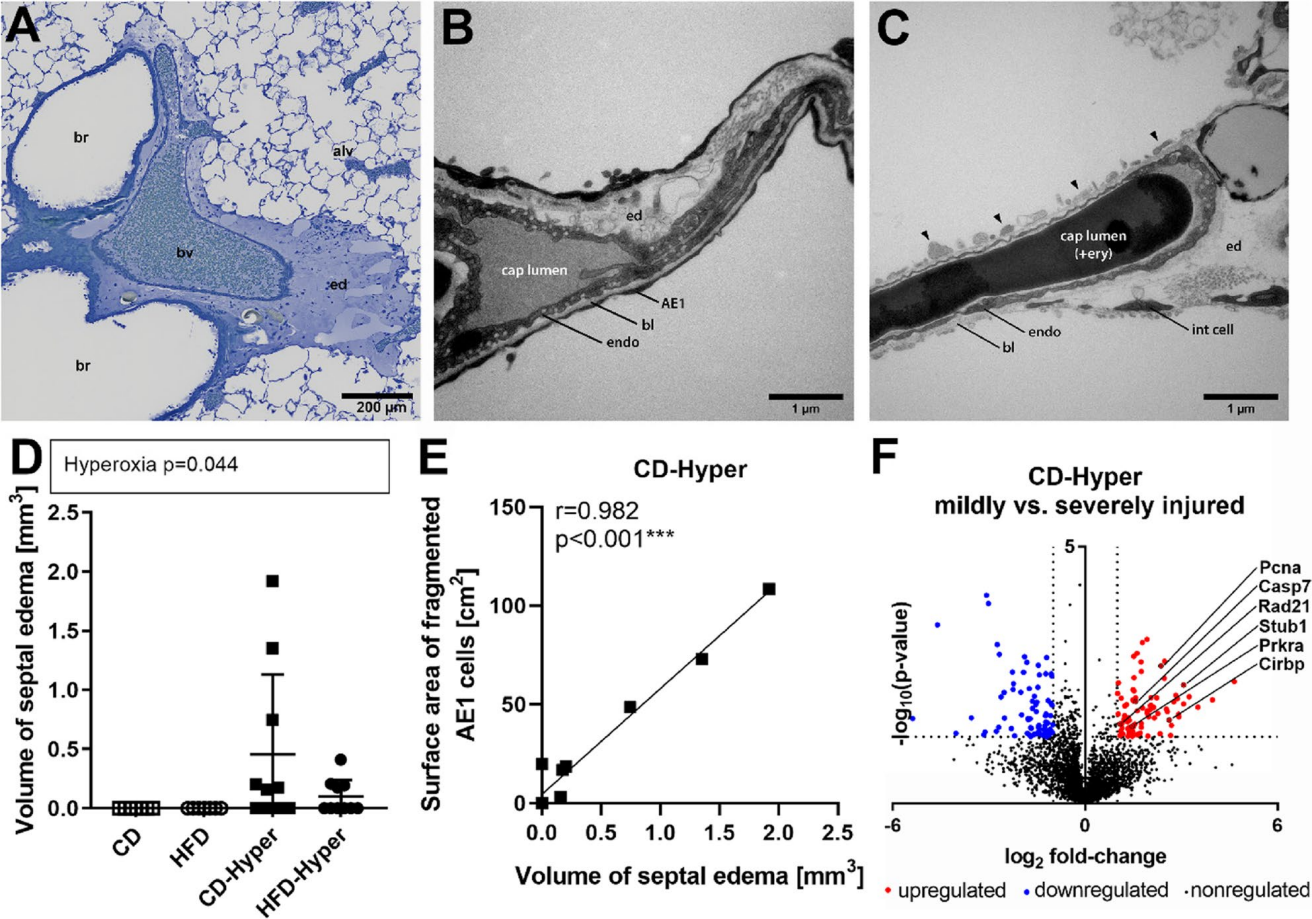
Edema formation in response to hyperoxia. **A** Representative light microscopic image of peribronchovascular edema (HFD-Hyper animal shown); br, bronchus; bv, blood vessel; alv, alveoli; ed, edema fluid. **B**, **C** Representative electron microscopic images of septal edema in intact septa (**B**; CD-Hyper animal shown) or next to fragmented AE1 cells (**C**; CD-Hyper animal shown); AE1, AE1 cell; endo, endothelial cell; int cell, interstitial cell; bl, basal lamina; ed, edema fluid; cap lumen, capillary lumen; +ery, with erythrocyte; arrowheads indicate fragmented AE1 cells. **D** Volume of septal edema; data are presented as individual values, group means and standard deviations are indicated; CD *n* = 7, HFD *n* = 7, CD-Hyper *n* = 10, HFD-Hyper *n* = 10; statistics: 2 W-ANOVA, significant *p*-values are shown above dot plot. **E** Correlation of surface area of fragmented AE1 cells with edema volume in CD-Hyper group, *n* = 10; linear regression indicated as line; statistics: Pearson correlation analysis, Pearsons *r* and *p*-values shown. **F** Volcano Plot for CD-Hyper animals with severe edema vs. other CD-Hyper animals, dashed lines represent (i) -log10 *p* > 1.301 and (ii) log2-fold change > 1.0 or < 1.0; proteins beyond these thresholds are color-coded in blue for reduced abundance (77 proteins) and in red for increased abundance (85 proteins), single proteins are highlighted and labelled with their names

Hyperoxia exposure induced peribronchovascular edema in some of the animals (Fig. 6A). Moreover, septal edema was detected as electron lucent areas in the interstitial space, either in intact septa (Fig. 6B) or colocalizing with AE1 cell fragmentation (Fig. 6C). The volume of septal edema was higher in CD-Hyper compared to HFD-Hyper (Fig. 6D), although the high inter-individual variance has to be taken into account here. The extent of septal edema significantly correlated with the observed AE1 cell injury in CD-Hyper (Fig. 6E) but not in HFD-Hyper (Fig. S6). In view of the bimodal edema response observed in the CD-Hyper group, a direct proteome comparison was performed between severely injured and mildly to non-injured animals (Fig. 6F). This analysis revealed reduced abundances of the mitochondrial proteins Tomm20 (mitochondrial import receptor subunit TOM20 homolog) and thioredoxin reductase 2, alongside increased levels of stress- and cell damage–associated proteins in severely injured animals compared with less affected animals.

## Discussion

This study demonstrates that diet-induced obesity has a significant impact on hyperoxia-related acute lung injury, particularly on the structural level. Morphological hallmarks of HALI including fragmentation and loss of AE1 cells as well as septal edema were significantly mitigated in obese mice. This was accompanied with preserved adipose tissue reserves in the HFD-Hyper group. Moreover, protein expression patterns indicated a partially diet-specific pulmonary adaptation to high oxygen exposure. These differences did not lead to improved functional parameters such as blood oxygenation or Horowitz index in the acute phase but point to a beneficial impact on regeneration.

Given the high prevalence of obesity, also more and more patients admitted to ICUs present with obesity, with rates between 28% and 36% [36–38]. Obesity is related to physical, metabolic, and molecular alterations across multiple organ systems, resulting in a higher risk for specific diseases including ARDS [39]. However, several studies report better outcome and lower mortality rates in obese ARDS patients, indicating potentially protective effects of obesity [16, 17, 40]. This “obesity paradox” is highly debated, and some researchers argue that it may be an artifact of methodological and statistical bias [20, 41]. Confounding variables such as age, smoking status, comorbidities and the severity or duration of the underlying disease may influence the observed outcomes. Furthermore, obese patients may receive more intensive medical surveillance and earlier interventions, potentially skewing survival statistics in favor of obese individuals. Another significant limitation is the reliance on BMI as a surrogate marker for adiposity since BMI does not account for body composition differences (e.g. muscle mass versus fat mass, fat distribution) [42]. Consequently, individuals with the same BMI may have markedly different health risks. It is currently unclear whether a biological basis for an obesity paradox exists.

Since the interpretation of clinical data is complicated by the methodological and/or statistical problems mentioned above, animal models are useful because they allow for standardization of experimental factors and environmental conditions. It has been shown in various animal species that exposure to high oxygen concentrations causes lung alterations which mimics the clinical and pathological features of acute lung injury in ARDS patients [2, 21]. In the HALI model used here (mice, FiO_2_ = 0.9 for 72 h) we were able to show that, under comparable and controlled conditions, diet-induced obesity had a measurable mitigating effect on hyperoxia-induced epithelial cell damage.

Alveolar epithelial cells form the airside of the septa and are first exposed to high FiO_2_. Therefore, fragmentation of AE1 cells with denudation of the basement membrane is a frequent consequence in hyperoxia [43]. This AE1 cell damage was present in 6 of 10 hyperoxic CD-fed mice, demonstrated by increased surface areas which were denuded or covered by fragmented AE1 cells. This was accompanied by septal edema formation in the affected animals. Moreover, AE2 cell numbers were also decreased. This hyperoxic epithelial cell injury was significantly alleviated in obese mice, resulting in reduced damaged and higher intact AE1 cell surface areas, reduced edema formation and higher AE2 cell numbers in HFD-Hyper compared to CD-Hyper. Moreover, total AE1 cell volumes were increased in the HFD-Hyper group, indicating differentiation of AE2 cells into AE1 cells. AE2 cells are epithelial progenitor cells which proliferate and replenish themselves and, after differentiation, also AE1 cells. Thereby AE2 cells are essential for alveolar repair and regeneration after lung injury [44, 45]. This regenerative function of AE2 cells appeared to be improved in hyperoxic HFD-fed mice, indicating an obesity-related positive effect. One conceivable explanation behind this observation are the higher lipid reserves, which may have enabled obese animals to better supply cell membrane components as well as energy. Moreover, cellular pathways uniquely regulated in HFD-Hyper animals included catabolic and biosynthetic processes indicating a metabolically active phenotype, that was not induced in CD-Hyper. In vitro studies indicate that both the glucose consumption and lactic acid production increase under hyperoxia exposure in alveolar epithelial cells, while the ATP production decreases [46]. The higher amounts of substrates for beta-oxidation in hyperoxic HFD-fed animals may have made the cells more adaptable and resistant to the cellular stress caused by hyperoxia. This is supported by the induction of cellular stress response-related pathways in CD-Hyper, and adaptive pathways such as protein folding and cytoplasmic translation in HFD-Hyper.

Diet-related differences in protein levels under hyperoxia included a higher abundance of the extracellular matrix glycoprotein fibronectin 1 in HFD-Hyper compared to CD-Hyper. Fibronectin 1 was shown in vitro to promote wound healing by alveolar epithelial cells and to stimulate alveolar epithelial barrier formation [47–49]. Moreover, recent studies demonstrated that exogenous administered fibronectin alleviated symptoms of acute lung injury in mice, including diminished edema formation and reduced inflammation and oxidative stress [50, 51]. In obesity, plasma fibronectin concentrations are increased [52, 53], thus, increased fibronectin amounts in HFD-Hyper may have contributed to the observed improved epithelial regeneration.

Furthermore, haptoglobin levels were elevated in the lungs of HFD-Hyper animals compared to CD-Hyper. Haptoglobin binds toxic cell-free hemoglobin, which is frequently detected in ARDS patients with sepsis [54]. Cell-free hemoglobin contributes to organ injury through several mechanisms, including damage to the vascular endothelium and nitric oxide scavenging [55], activation of neutrophils [56], and oxidative stress [57]. Consistently, reduced function of a genetic haptoglobin variant has been associated with increased susceptibility to ARDS in both animal models and humans [58] and overexpression of haptoglobin resulted in attenuated blood-induced lung injury and inflammation in transgenic mice [59]. Haptoglobin is an acute-phase protein that is upregulated in both inflammation and obesity. It is abundantly expressed in white adipose tissue, and circulating haptoglobin levels correlate positively with body mass index (BMI) [60, 61]. Although haptoglobin appears to exert adverse effects on metabolic function in obesity [62], the results presented here suggest that it may play a protective role in the context of HALI-induced lung injury.

The antioxidant agent selenoprotein P was also elevated in HFD-Hyper. Patients with severe lung injury typically exhibit significantly lower levels of selenoprotein P and research indicates that selenium supplementation in ARDS patients can improve oxygenation efficiency and survival trends [63, 64], indicating a beneficial effect on HALI in HFD-Hyper.

Notably, some animals in the CD-Hyper group exhibited more severe lung injury than others. Proteome comparisons revealed that proteins associated with cellular stress and cell damage were more strongly expressed in severely injured animals than in less affected animals. Hyperoxia induces reactive oxygen species that can damage genomic DNA. Cells may either repair this damage or undergo apoptosis or necrosis [65]. Consistently, severely injured animals showed increased levels of the DNA repair proteins Pcna (proliferating cell nuclear antigen) and Rad21 (double-strand-break repair protein Rad21 homolog), but also higher abundances of the apoptosis effector protein Caspase-7 and Prkra (interferon-inducible double-stranded RNA-dependent protein kinase activator A), which has been shown to promote cell death under stress conditions, including oxidative stress [66]. In mouse models of HALI, the proportion of apoptotic cells correlates with the severity of lung injury [67]. Together, these findings suggest an imbalance between DNA repair mechanisms and pro-apoptotic signaling, consistent with more severe epithelial damage observed in these animals.

Despite the alleviating effect of obesity on structural parameters, functional impairments such as reduced blood oxygenation and pulmonary stiffening were comparable in both hyperoxic groups. This suggested that in the acute phase, i.e. within the hyperoxic environment, obesity did not confer any advantage. However, due to cellular improvements mentioned above, obesity could augment recovery and thus contribute to the lower long-term mortality rates observed in obese ARDS patients [40].

The observed drop in oxygen levels is likely due to the fact that adaptation to normoxia had not yet been achieved. The time between the removal of the individual animal from the hyperoxia chamber and the blood gas measurement was approximately 15 min. Thus, the respiratory depression caused by hyperoxia exposure was still present; this ‘respiratory plasticity’ has been described previously [68, 69].

The pulmonary stiffening indicated by the reduced respiratory system compliance corresponds to findings in ARDS patients. These patients show severely decreased respiratory system compliance, which is attributed to alveolar edema and inflammatory processes leading to surfactant inactivation and atelectasis of distal airspaces [70, 71].

Previous studies on obesity-related influences on different HALI models have revealed controversial results. Consistent with our data, a study on HALI (FiO_2_=1.0, 84 h) reported improved survival and reduced circulating inflammatory cytokines and lung edema in obese leptin-resistant hyperphagic mice [23]. In contrast, HFD-feeding for 12 weeks resulted in reduced survival and more severe lung damage measured by qualitatively assessed morphological alterations and more TUNEL-positive cells upon HALI (FiO_2_>0.9, survival or 48 h) [22]. Our study differs from previous work in the feeding duration which was significantly longer than in other studies. This feeding protocol was shown before to allow for manifestation of obesity-related alterations comparable to human obesity, including increases in various white and brown adipose tissue masses, hypercholesterolaemia as well as elevated insulin concentrations, insulin resistance and delayed glucose clearance in oral glucose tolerance assays indicating a pre-diabetic state [13, 14, 25, 26, 72]. Moreover, this study utilized a range of different methods to assess lung damage including quantitative morphometry in contrast to determination of e.g. BALF proteins or wet to dry ratio.

It was reported before that male mice develop more severe lung injury and inflammatory activation in response to HALI [73], thus only male mice were used in the experiments reported here. As a consequence, gender differences regarding the results cannot be excluded, what can be considered as a limitation of the study. Moreover, the proteome analysis was carried out with bulk lung tissue, therefore differences in protein abundances cannot be traced back to individual cell populations. Furthermore, follow-up experiments are needed to investigate the regeneration of the alveolar epithelium over longer recovery periods and to conclusively determine the long-term impact of HFD on epithelial repair mechanisms.

## Conclusions

Taken together, diet-induced obesity prevented from body fat depletion and alleviated epithelial cell damage and edema formation under hyperoxic conditions. This supports a biological basis for an obesity paradox that may be facilitated by increased metabolic reserves. Future studies are warranted to explore further underlying mechanisms and transfer these findings to the human situation to refine therapeutic strategies in obese ARDS patients.

## Supplementary Information

Supplementary Material 1.

Supplementary Material 2.

## Data Availability

All data generated or analysed during this study are included in this published article and its supplementary information files.

## Abbreviations

ABB: Air-blood barrier
AE1: Alveolar epithelial cell type 1
AE2: Alveolar epithelial cell type 2
ARDS: Acute respiratory distress syndrome
BMI: Body mass index
CD: Control diet
CD-Hyper: Control diet with hyperoxia exposure
FiO_2_: Inspired oxygen fraction
HALI: Hyperoxic acute lung injury
HFD: High fat diet
HFD-Hyper: High fat diet with hyperoxia exposure
ICU: Intensive care unit
LM: Light microscopy
PCA: Principal component analysis
PEEP: Positive end expiratory pressure
ROS: Reactive oxygen species
SDS-PAGE: Sodium Dodecyl Sulfate - Polyacryamide Gel Electrophoresis
SURS: Systematic uniform random sampling
TEM: Transmission electron microscopy
2W-ANOVA: Two-way ANOVA
