# Supplementary material for: Diet-induced obesity alleviates epithelial damage in hyperoxic acute lung injury (HALI) in mice
Source: Respir Res. 2026 Apr 9;27:189. doi: 10.1186/s12931-026-03663-w (PMC13122894; doi:10.1186/s12931-026-03663-w)
Supplement: Supplementary file 1 — Supplementary Material 1. [file 12931_2026_3663_MOESM1_ESM.pdf]

Supplemental table S12. Results of 2-Way ANOVA

Parameter	Diet	Hyperoxia	Interaction
Body weight difference [%, W29 vs. finals]	0.251	**<0.001**	0.208
Final body weight [g]	**<0.001**	**<0.001**	**0.034**
Fat, epididymal [g]	**<0.001**	**<0.001**	*0.071*
Fat, retroperitoneal [g]	**<0.001**	**0.002**	**0.014**
Fat, interscapular [g]	**<0.001**	0.184	0.353
Horowitz index [mmHg, pO_2/F_iO_2]	0.845	**<0.001**	0.398
Blood pH	0.617	0.992	0.678
Bicarbonate [mmol/l]	**0.004**	**<0.001**	*0.093*
Total carbon dioxide [mmol/l]	**0.002**	**<0.001**	*0.073*
Oxygen saturation [%]	0.894	**0.010**	0.541
Oxygen partial pressure [mmHg]	0.746	**0.006**	0.361
Carbon dioxide partial pressure [mmHg]	0.201	**<0.001**	0.142
Quasi-static compliance [ml/cmH_2O]	0.423	**<0.001**	0.949
Hysteresis [ml x cmH2O]	0.607	**0.011**	0.408
Left lung volume [mm^3]	**0.032**	0.101	0.274
Septal volume [mm^3]	0.320	0.707	1.000
Ductal airspace volume [mm^3]	*0.051*	**0.003**	0.413
Alveolar airspace volume [mm^3]	0.108	0.335	0.415
Septal surface area [cm^2]	*0.095*	*0.095*	0.132
Septal thickness [µm]	0.808	0.161	0.147
Mean linear intercept length [µm]	0.345	0.220	0.929
Surface area of intact AE1 cells [cm^2]	*0.062*	0.989	*0.096*
Surface area of fragmented AE1 cells [cm^2]	*0.059*	**0.041**	*0.059*
AE1 cell volume [mm^3]	**0.006**	0.281	**0.042**

AE2 cell volume [mm^3]	0.830	**0.010**	0.191
AE2 cell number	*0.065*	**<0.001**	0.101
Thickness of AE1 cells [µm]	**0.046**	0.919	0.183
Thickness of septal interstitium [µm]	0.720	0.286	*0.057*
Thickness of endothelial cells [µm]	**0.027**	**0.013**	0.118
Thickness of air-blood barrier [µm]	*0.073*	*0.088*	0.140
Volume of septal edema [mm^3]	0.185	**0.044**	0.185

Exact p-values are given. Significant p-values ($p<0.05$) are shown in bold, p-values with a tendency to significance ($0.05 < p < 0.1$) are shown in italic letters.

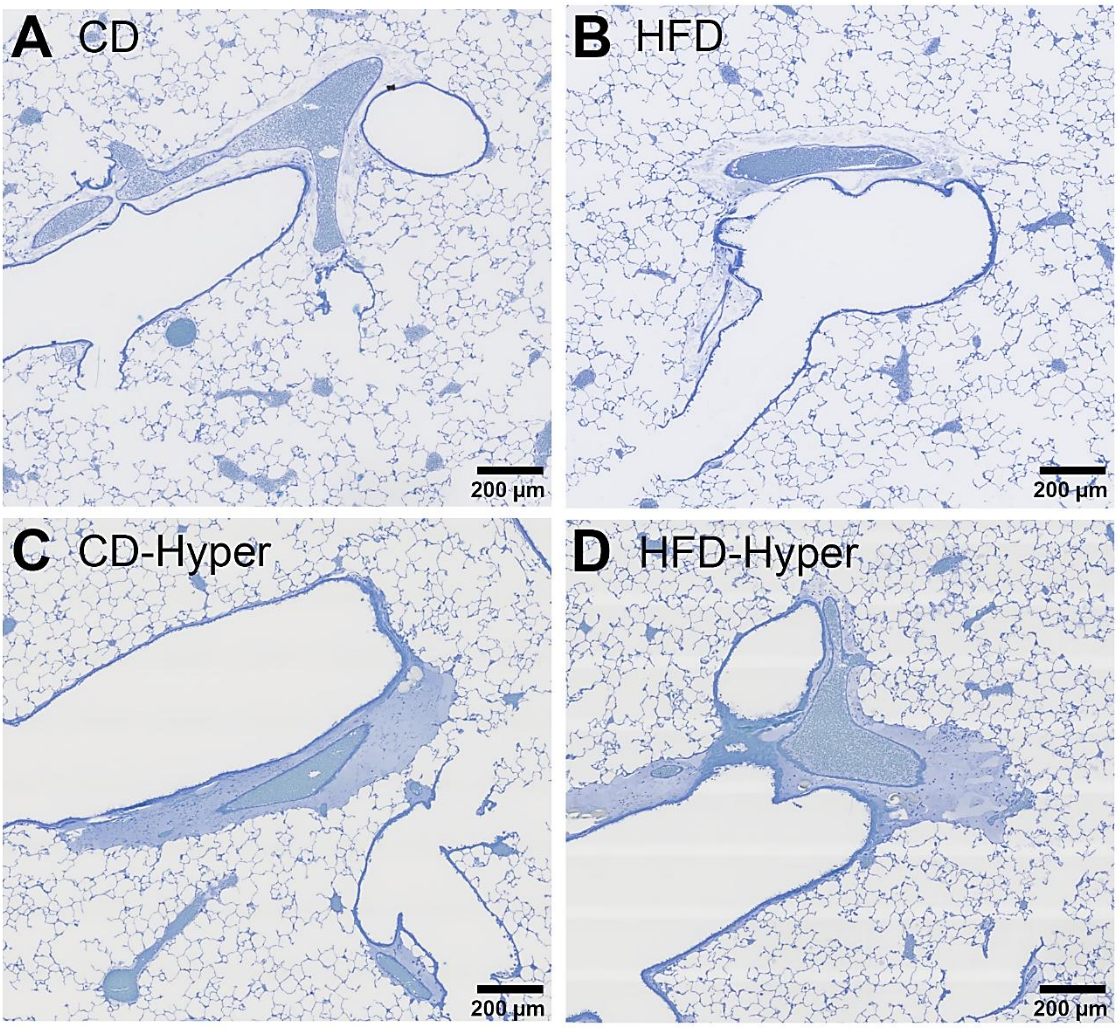

Fig. S1: Images of peribronchovascular region of experimental groups at light-microscopical level. Images of hyperoxic groups show peribronchovascular edema. Please note that regions without edema were also present in the hyperoxic groups, therefore a detailed morphometric quantification of edema was performed.

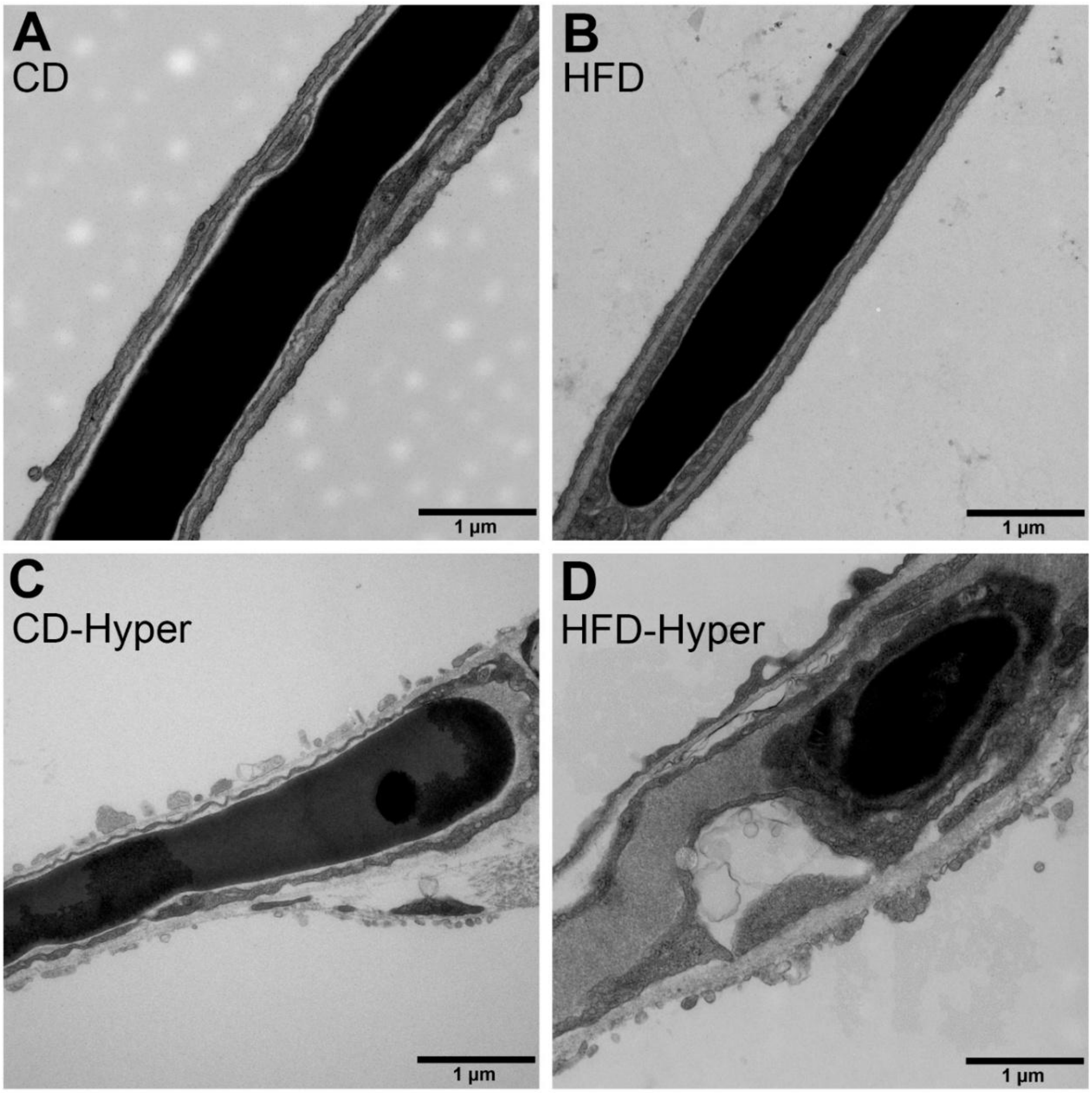

Fig. S2: Images of lung structure of experimental groups at electron-microscopical level. Images of hyperoxic groups show epithelial cell damage and septal edema. Please note that intact septa were also present in the hyperoxic groups, therefore a detailed morphometric quantification of epithelial damage and edema was performed.

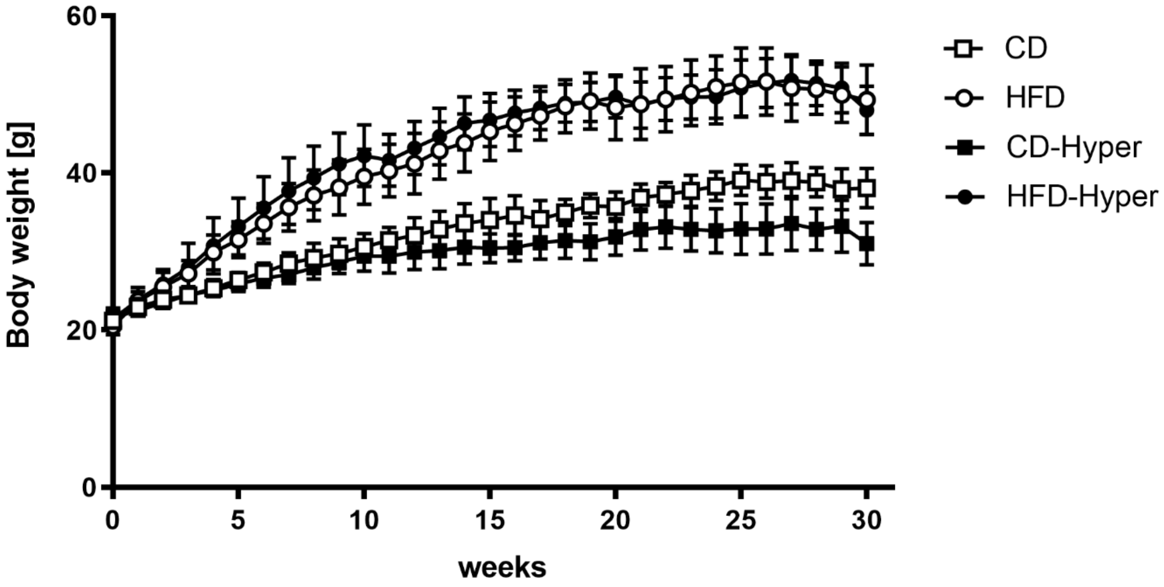

Fig. S3: Body weight. Symbols reflect group means, standard deviations are indicated.

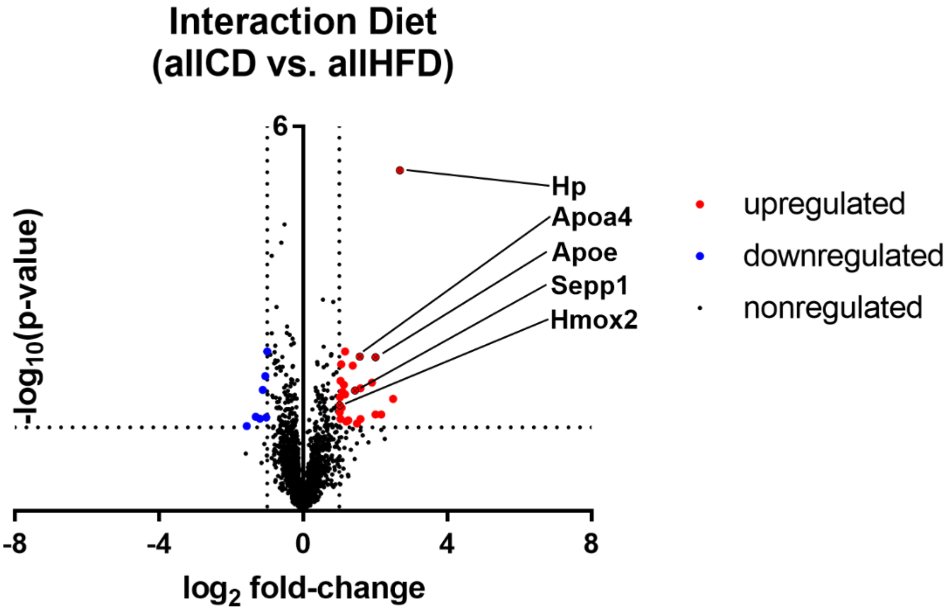

Fig. S4: Effect of factor diet on differentially expressed pulmonary proteins. Volcano plots, dashed lines represent i) -log10 p>1.301 and ii) log2-fold change >1.0 or <1.0; proteins beyond these thresholds are color-coded in blue for reduced abundance and in red for increased abundance; single proteins are highlighted and labelled with their names.

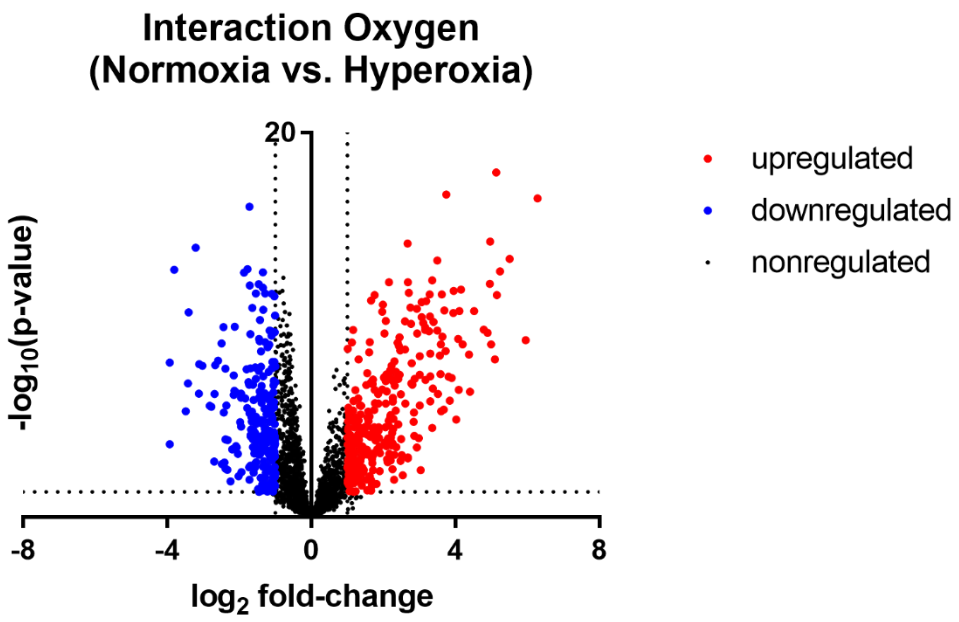

Fig. S5: Effect of factor oxygen on differentially expressed pulmonary proteins. Volcano plots, dashed lines represent i) -log10 p>1.301 and ii) log2-fold change >1.0 or <1.0; proteins beyond these thresholds are color-coded in blue for reduced abundance and in red for increased abundance.

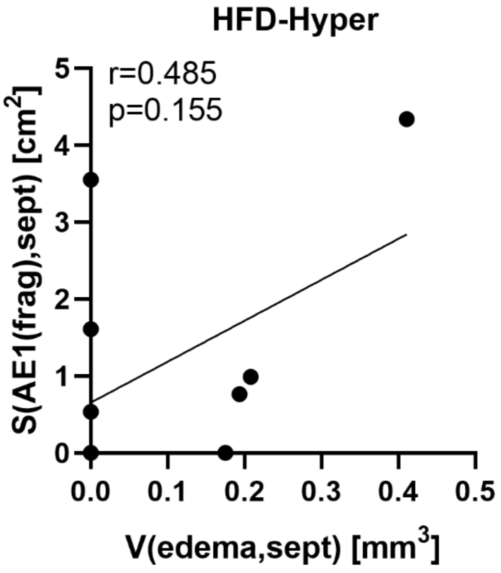

Fig. S6: Correlation analysis of septal edema and alveolar epithelial damage in HFD-Hyper. Correlation of surface area of fragmented AE1 cells with edema volume in HFD-Hyper group, n=10; linear regression indicated as line; statistics: Pearson correlation analysis, Pearsons r and p-values shown.
